# Rediscovering digitules in Aphidomorpha and the question of homology among Sternorrhyncha (Insecta, Hemiptera)

**DOI:** 10.3897/zookeys.683.10100

**Published:** 2017-07-06

**Authors:** Mark A. Metz, Douglass R. Miller, Aaron M. Dickey, Gary R. Bauchan, Ronald Ochoa, Michael J. Skvarla, Gary L. Miller

**Affiliations:** 1 USDA, ARS, Systematic Entomology Laboratory, c/o Smithsonian Institution, National Museum of Natural History, E-524, P.O. Box 37012, MRC 168, Washington, DC 20013-7012, USA; 2 USDA, ARS, Systematic Entomology Laboratory, Henry A. Wallace Beltsville Agricultural Research Center, 10300 Baltimore Avenue, Building 005, Beltsville, Maryland 20705, USA; 3 Florida State Collection of Arthropods, Florida Department of Agriculture and Consumer Services, Gainesville, Florida, 32614, USA; 4 US Meat Animal Research Center, P.O. BOX 166 (State Spur 18D)/USDA-ARS-PA-MARC, Clay Center NE 68933, USA; 5 USDA, ARS, Electron and Confocal Microscopy Unit, Henry A. Wallace Beltsville Agricultural Research Center, 10300 Baltimore Avenue, Building 012, Beltsville, Maryland 20705, USA; 6 Department of Entomology, University of Maryland, 4112 Plant Sciences Building, College Park, MD 20742, USA

**Keywords:** Aphididae, Phylloxeridae, Adelgidae, Coccoidea, Coccidae

## Abstract

We explore and expand on the morphological term digitule. The term was originally proposed for toe-like setae on a species of *Phylloxera* Boyer de Fonscolombe, 1834 (Hemiptera, Sternorrhyncha, Aphidomorpha) by Henry Shimer, an American naturalist. While it is standard terminology in scale systematics (Hemiptera, Sternorrhyncha, Coccidomorpha), the term digitule was ignored by aphid specialists despite being the original taxon for which the term was described. Similar setae occur on many arthropod groups, so the homology is poorly understood even within any superfamily of Hemiptera. We provide the etymology of the term, a proposed explanation for why it was used among scale taxonomists and not aphid taxonomists, and discuss briefly options to progress beyond the confusion between terminology for morphology and homology in Sternorrhyncha.

## Introduction

The hemipteran suborder Sternorrhyncha includes the aphids (Aphidomorpha), scale insects (Coccidomorpha), whiteflies (Aleyrodomorpha), and the psyllids or jumping plant lice (Psyllidomorpha) (Carver et al. 1991). Scale insects often have one or more specialized setae on or near the terminus of the leg called digitules. Most scale taxonomists use the term based on setal shape and position on the leg (i.e., it is differentiated from nearby setae in some way, most often enlarged and with an enlarged apex and is near or at the apex of the leg) (Williams 1985, Hodgson 1994, Hardy et al. 2008). If a seta occurs on the claw it is considered to be a digitule regardless of the shape. If the setae on the dorsoapical position of the tarsus are conspicuously larger than the other leg setae they also are treated as digitules. There have been a few inconsistencies in this usage, but they are the exception rather than the rule. Setae with similar shape and position also occur in some species of Aphidomorpha, and differentiated setae occur at or near the apex of the leg in psyllids (pulvillus) (Ossiannilsson 1992) and whiteflies (paronychium) (Gill 1990), but they are not called digitules in these groups. And similar looking setae are found in some mites (Acari) and springtails (Collembola) and are sometimes called digitules (MacGillivray 1923). The lack of congruence among anatomy, morphology, and homology is not uncommon in taxonomy and is often deeply entrenched after years of tradition and unresolved evolutionary relationships among taxa. We had the opportunity to explore this issue with the term digitule after rediscovering the presence of a similar structure in a presumably undescribed species of the genus *Phylloxera* Boyer de Fonscolombe, 1834 (Insecta, Aphidomorpha, Phylloxeridae).

The genus *Phylloxera* includes 51 valid species; the majority of which were described feeding on *Carya* Nuttall, 1818 in North America (Pergande 1904, Favret 2017). All *Phylloxera* are herbivores with sucking mouthparts and induce the formation of galls on the plant tissues on which they feed. The galls are presumed to be species-specific in their shape, size, color, etc., so species identification relies heavily on host plant and gall morphology. The family Phylloxeridae is part of a monophyletic Aphidomorpha, which includes the superfamilies Phylloxeroidea and Aphidoidea (i.e., aphids), and Aphidomorpha has a sister-group relationship with the Coccidomorpha (scale insects) within the sternorrhynchan suborder of Hemiptera (Gullan and Cook 2007). Scale insects are more diverse and richer in morphology than the other sternorrhynchan groups, taxonomists have relied on these morphological characters to diagnose taxa, and family group relationships among Coccidomorpha are more resolved (Hodgson and Hardy 2013). It's possible that the historical reliance on gall morphology in *Phylloxera*, differing with the use of specimen morphology in scale insects, has contributed to the use and lack thereof of the term digitule in each taxon. We examine that possibility in the purview of the term's etymology and briefly discuss the issue of homology.

### History of the term digitule

All of the dictionaries and texts on entomology we checked define digitule with a similar rendition of the same phrase, and are possibly or known to be non-independent repetitions. For example:

The Dictionary of Entomology (Jardine 1913):

*Digitules. - Appendages usually present on the feet of the Coccidae, either broadly dilated or in the form of knobbed hairs. (From L. digitus)*

External Insect-anatomy: A Guide to the Study of Insect Anatomy and an Introduction to Systematic Entomology (MacGillivray 1923, p. 247):

*Digitules. - The distal end of the distal tarsal segment and the proximal part of the claws may bear long slender setae that are clavate at the distal end. These setae are known as digitules, also as tenent hairs or empodial hairs. . . . The digitules are of more general occurrence in minute insects like the collembolans and the males of coccids*.

The Dictionnaire des Termes D'Entomologie (Séguy 1967):

*digitule n. m. Appendice des pattes des Coccides qui peut être une soie dilatée ou terminée par un bouton; soie adhésive; soie empodiale. - Digitule unguéal: chète ou cil placé sur les ongles*.

The Torre-Bueno Glossary of Entomology (Nichols1989):

*digitule(s), in Coccoidea (Hemiptera: Sternorrhyncha), a pair of normally capitate setae at the inner base of the tarsal claws and at the outer distal margin of the tarsus (T-B, after MacGillivray; Kosztarab and Kozár)*.

A Dictionary of Entomology (Gordh 2001, 2011):

*DIGITULE Noun. (Latin, digitus = finger. Pl., Digitules.) 1. Coccidae: Appendages of the feet that may be broadly dilated or knobbed Setae. 2. Tenent hairs; empodial hairs (MacGillivray)*.

**Figure 1–6.**
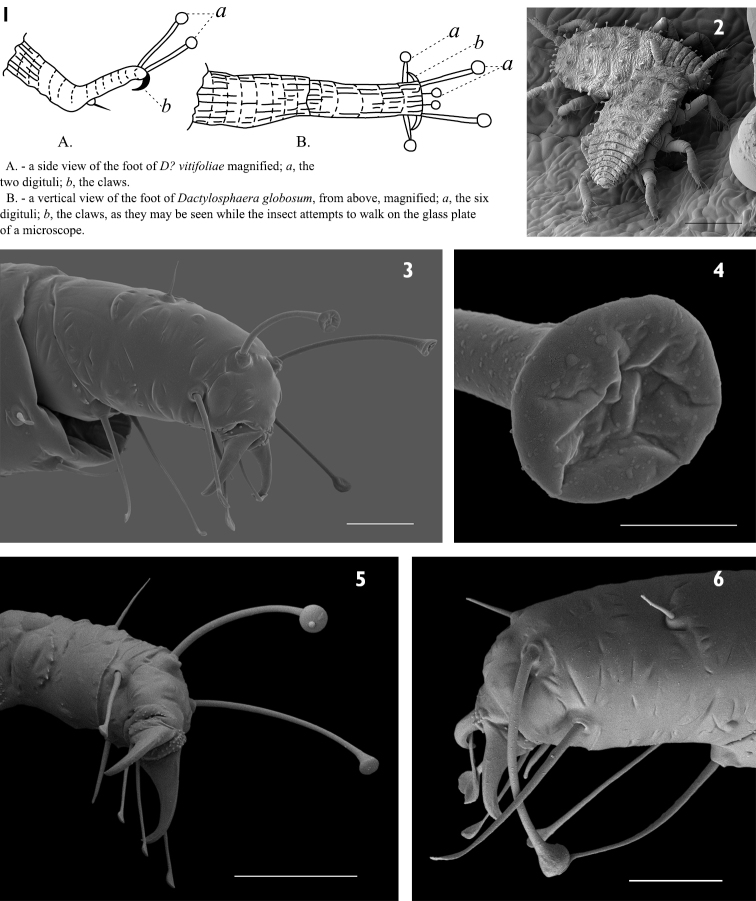
**1** Reproduction of Shimer’s (1867a) original illustration indicating what he considered digitules **2** *Phylloxera* in situ showing leg and feet position **3** Hind leg of *Phylloxera* showing all digitules **4** Close up of dorsal digitule from Figure 3. Note how the ventral surface appears membranous and collapsed **5** Hind leg of *Phylloxera* showing dorsal digitules with expanded ventral surfaces **6** Front left leg of *Phylloxera* showing anterior seta is not a digitule. Scale bar 10 μm. Scale bars: (**2, 6**)100 μm; (**3**) 10 μm; (**4**) 2 μm; (**5**) 20 μm.

All the above descriptions associate the term digitule with Coccidae/Coccoidea (=Coccidomorpha), which became part of that taxon's common vernacular by the late 19^th^ to early 20^th^ century based on descriptions of new taxa and other taxonomic works (e.g., Signoret 1872a, 1872b, 1873, 1874; Targioni Tozzetti 1875, Haller 1880, Atkinson 1886, Maskell 1887, Ashmead 1891, Berlese 1893, Cockerell 1893, Hunter 1899a, 1899b, 1900, 1902, Hempel 1900a, 1900b, 1901a, 1901b). A single origin of the term was unclear until MAM recently uncovered a paper by Henry Shimer (1867a), an American naturalist, wherein he described a new family and genus for a gall-forming hemipteran, *Dactylosphaera globosum* (Shimer, 1867), on *Carya glabra* (Miller), which is currently a valid species in the genus *Phylloxera*. This appears to be the first incidence of the term in the literature (Fig. 1):

Tarsi composed of one joint, terminated by two claws, and from two to six digituli.*

*I suggest this name, digituli, from the Latin digitulus, a small finger or toe, for these remarkable organs; it appears to me appropriate, because they are arranged around the foot somewhat like the toes of an animal.

The adoption of digitule to describe toe-like setae at the terminus of the legs in Coccidomorpha instead of Aphidomorpha seems to be historical and serendipitous. The following year, Shimer (1868) used digituli again, referencing his 1867 work therein, when he described a new family, Lepidosaphidae, and genus, *Lepidosaphes*, for the species *Coccus conchiformis* Gmelin, 1790, which was then and is currently classified as a species of scale-insect. Two prominent specialists on Coccidomorpha were Victor Antoine Signoret (1816–1889) of Paris and Adolfo Targioni-Tozzetti (1823–1902) of Florence who recorded in their own articles that they were aware of the other's work. Signoret (1872a) cited Shimer as the source when he first used the term (as digituli), and subsequent works by both authors referred to these structures as digituli or digitules. Their prominence in this field of study presumably led to the community accepting this term for the large part. The term has such wide acceptance that even homology is assumed, as acuminate setae in the same position are still called digitules by some coccidomorph specialists (e.g., Williams and Hodgson 2013).

In contrast, the scientists with the most impact studying Phylloxeridae during that time i.e., Asa Fitch (1809–1879), Charles Valentine Riley (1843–1895), Theodore Pergande (1840–1916), and Benjamin Dann Walsh (1808–1869), were Americans who focused primarily on gall morphology, the biologies of the insects, and the control of pests on commodities. Pergande (1904) used digituli in quotes signifying that the source was from a posthumous or silent coauthors' writings (i.e., Riley was deceased and Dreyfus had to postpone his involvement with *Phylloxera* indefinitely due to “financial and other troubles.”). These authors were less invested in the detailed morphology of the insects themselves throughout their writing. We assume this is a cultural phenomenon rather than a limitation in technology as other investigators of the period were able to visualize these fine structures (e.g., Signoret also published in this family and was a primary investigator of grape *Phylloxera* with Riley (Sorensen et al. 2008)). Of note, Packard (1898) used the term tenent hair instead of digitule, a listed source in MacGillivray's textbook (1923), and Oestlund (1887), perhaps the greatest influence on American aphid classification at the time, described these structures as “two capitate hairs as usually in the genus.” This seems to have carried through modern usage in Aphidomorpha (e.g., Foottit and Richards 1993, ventral setae on the apical sclerite (planta) of the pretarsus, Ar = plantar setae).

## Methods

AMD collected galls of presumably undescribed species of *Phylloxera* feeding on *Carya floridana* Sargent, 1913 at two sites in Saint Lucie County, Florida, USA from late February to early March 2012, 2013, and 2015. In the lab we sliced off the top of galls and observed specimens in situ or removed specimens from their galls and secured them to 15 mm × 30 mm copper plates using ultra smooth, round (12 mm diameter), carbon adhesive tabs (Electron Microscopy Sciences, Inc., Hatfield, PA, USA). We then followed the technique of Fisher et al. (2011) as follows. We froze specimens conductively, in a Styrofoam box, by placing the plates on the surface of a pre-cooled (-196°C) brass bar whose lower half was submerged in liquid nitrogen (LN2). After 20–30 seconds, we transferred the holders containing the frozen samples into a Quorum PP2000 cryo-prep chamber (Quorum Technologies, East Sussex, UK) attached to an S-4700 field emission scanning electron microscope (Hitachi High Technologies America, Inc., Dallas, TX, USA). The specimens were etched inside the cryotransfer system to remove any surface contamination (condensed water vapor) by raising the temperature of the stage to -90°C for 10–15 min. Following etching, we lowered the temperature of the stage inside the cryo-transfer system to -130°C, and coated the specimens with a 10 nm layer of platinum using a magnetron sputter head equipped with a platinum target. Finally, we transferred specimens to a pre-cooled (-130°C) cryostage in the LT-SEM for observation with an accelerating voltage of 5 kV to view the sample and we captured images using a 4 pi Analysis System (Durham, NC, USA).

We also obtained color images and videos of specimens in situ using a Hirox KH-7700 Digital Microscope (Hackensack, NJ) with a MXG-5040RZ lens to assess locomotion. The digital microscope has a motorized stage which allows the capture of several images at 1600×1200 pixels per frame with varying degrees of focus, which were compressed together to develop an image where all fields of view are in focus. We recorded video at 800×600 pixels per frame at 15 frames per second. We collected this imagery before freezing as reference material for observations made with the LT-SEM. MAM reproduced Shimer's (1867a) original illustration of digitules and composed figures with the vector drawing application INKSCAPE and made minor adjustments to the LT-SEM photographs with the photo-editing application GIMP.

## Results

Three distinct *Phylloxera* gall morphologies occurred at the sites: one Pergande (1904) group IV type and two Pergande (1904) group II fleshy leaf gall species most similar to *P. rimosalis* Pergande, 1904 and *P. caryaeglobuli* Walsh, 1863. The specimens had egg and adult characteristics most similar to *P. rimosalis*, as originally described by Pergande (1904). AMD sent intact galls from Florida to GLM at the Systematic Entomology Laboratory.

Based on Shimer's (1867a) original figures (Fig. 1) and his description of the structures, we interpret his definition of digitule to be a specialized seta with an expanded tip at the apex of the leg. There are a total of eight setae we would call digitules and two setae that are not digitules on the tarsus of *Phylloxera* we examined. The ventral setae at the apex of the tibia are also digitule-like. The basitarsus (Fig. 3) has a single pair of digitules at the ventroapical margin. The distitarus has a single, acuminate seta in the middle of the dorsum; a single, thick, blunt seta ventromedially at the subapex; two pairs of digitules at the apicoventral margin; and one pair of digitules dorsally at the apex (Figs 3, 5). The foreleg is an exception to the pattern with the anterior seta dorsally at the apex of the distitarsus being acuminate rather than expanded at the tip (Fig. 6). The digitules on the ventral surface all have apices that are spatulate or otherwise expanded in one plane. The dorsal digitules have apices that are expanded radially forming hemispherical knobs that seem membranous such that in some views they are smoothly convex (Fig. 5) while in others they are roughly concave (Figs 3, 4). The digitules are the longest of all the tarsal setae, over twice as long as the acuminate seta on the dorsum of the distitarsus and nearly a third longer than the blunt seta at the subapex of the ventromedial margin of the distitarsus. The digitules of the distitarsus also extend beyond the apices of the tarsal claws.

Digitules seem pliable as they are commonly bent when in contact with the substrate even though in some images the substrate seemed soft enough to take an impression from the digitules and tarsal claws. We did not observe any consistency of position of digitules and claws in relation to specimen activity to make any determinations of their function, and could not indisputably determine that any specimens were in locomotion at the time of freezing. Most specimens with their feet in contact with substrate also had their rostrum embedded in the gall inner wall (Fig. 2), and among these the feet positions varied from leg to leg and specimen to specimen despite the specimens being in an assumed stationary position. In some instances the tarsal claw was plantar flexed so that the dorsal surface of the claw was in contact with the substrate. In others, the ventral surface of the tarsal claw was in contact with the substrate. Again, these positions were likely to occur at the same time on different legs within any one specimen.

## Discussion

Through attempts to observe the interaction of *Phylloxera* with their gall substrate, we saw interesting setae on the legs and, after careful mining of the literature, found the originally intended term for these structures. Ironically, the original intent was lost in history, and, unfortunately for Henry Shimer, there is no Principle of Priority for morphological terms! Re-examining these structures with modern equipment, however, did allow us to make some novel observations. At least in the *Phylloxera* we examined, ventral digitules are expanded in only one plane and dorsal digitules are expanded radially. Digitules seem to be pliable, as they often conformed to the gall surface. And while we could not confirm any association of these structures with locomotion, they seem unable to support any significant weight, so if they indeed impart some role in association with the substrate perhaps it is sensory or a form of adhesion. Though not called digitules among Aphidomorpha, we did conduct a limited survey of the group. There does not appear to be a correlation between the presence of digitules and the habit of forming a gall, or with any other cryptic behavior. Among Aphidomorpha, digitules occur at least in the genera *Anoecia* Koch, 1857; *Cerataphis* Lichtenstein, 1882; *Ceratoglyphina* van der Goot, 1917; *Ceratovacuna* Zehntner, 1897; *Colopha* Monell, 1877; *Dinipponaphis* Takahashi, 1962; *Eriosoma* Leach, 1818 (species formerly in *Georgiaphis* Maxson & Hottes, 1926); *Gharesia* Stroyan, 1963; *Glyphina* Koch, 1856; *Hamamelistes* Shimer, 1867b; *Hormaphis* Osten-Sacken, 1861; *Nipponaphis* Pergande, 1906; *Phylloxera* Boyer de Fonscolombe, 1834; *Phylloxerina* Börner, 1908; *Tamalia* Baker, 1920; and *Thelaxes* Westwood, 1840 at some life stage (Fottit and Richards 1993 and direct observation of specimens). While there are species among these genera that are gall-formers, many make only a pseudogall or do not form galls at all. Likewise, species among the genera *Cornaphis* Gillette, 1913; *Forda* von Heyden, 1837; *Kaltenbachiella* Schouteden, 1906; *Melaphis* Walsh, 1867; *Pachypappa* Koch, 1856; *Pemphigus* Hartig, 1839, *Thecabius* Koch, 1857; *Tetraneura* Hartig, 1841; and *Tiliphagus* Smith, 1965 make either a pseudogall or true gall and have no digitules (Foottit and Richards 1993 and direct observation of specimens). In essence we have come full-circle in terms of digitules in aphids. The structures were first discovered in aphids, we rediscovered them in aphids, but the term digitule is not used in aphids. So the question of digitule homology is not at issue in Aphidomorpha as it is in Coccidomorpha.

The use of the term digitule is prevalent in coccidomorph literature, and is confounded by the lack of distinction between shape and positional homology as we mentioned above. All taxa among Coccidomorpha are considered to have digitules, and all families except Ortheziidae and Stigmacoccidae have species with capitate setae, or setae with expanded apices in some form, in at least one life stage. The following are two examples to illustrate extreme differences in interpretation and not meant to be comprehensive. The recently diagnosed species *Arctorthezia helvetica* Kozár & Szita, 2015 (Ortheziidae) is described as having claw digitules, as do all the other species treated in that genus by the authors (Szita et al. 2015). These setae are undifferentiated from the setae on the remaining hind leg, but are called digitules because of their position ventrad on the base of the tarsal claw. The most distal setae dorsad on the basal tarsal segment, however, are not described as digitules despite having the same position as digitules from other coccidomorph taxa. These setae are undifferentiated from the remaining setae on that tarsal segment, or from most of the setae on the entire leg. In contrast, the species *Steingelia gorodetskia* Nasonov, 1908 and *Stomacoccus platani* Ferris, 1917 (Steingeliidae) have multiple (up to 10) setae with expanded apices located on all surfaces around the base of the tarsal claw. All are called digitules even though they do not share the ventrad position as digitules in other coccidomorph species. We assume these are considered digitules because of their shape rather than their position. Unlike the above authors' treatment in Ortheziidae, however, even fine, hair-like setae dorsad on the apex of the basal tarsal segment are considered digitules in other margarodoid Coccidomorpha (Hodgson and Foldi 2006). Even if these are rare exceptions among scale workers, defining and homologizing these setae uniformly across all of Sternorrhyncha remains a challenge.

So what, if anything, can we recommend to progress towards a stable solution? Testing homology across all of Sternorrhyncha through cladistic analysis is a far-reaching goal and certainly beyond the scope of this work. One possible course of action would be to treat the digitule as morpheme rather than homology (Vogt et al. 2010, Richter and Wirkner 2014). As a morpheme, the term digitule could be applied to those structures similar to what Shimer (1867a) originally observed (viz., setae on the terminus of the legs with expanded apices) to any taxon without assumption of homology. This provides a tool for description of an anatomical element with which others can then use for comparative anatomy and character transformation. This solution, however, would disrupt both the current consensus among scale workers to describe non-differentiated setae as digitules based on position and the complete lack of the term among aphid workers. Currently and ultimately, accurate communication without ambiguity between a taxonomist and the reader is the best course of action. So, we recommend fundamentally that sternorrhynchan taxonomist are clear on their usage or non-usage of the term digitule in light of this work. Because one thing that has become evidently clear is that originally digitules were not just for scales, but for aphids, too.
